# Une langue étrange

**DOI:** 10.11604/pamj.2015.22.93.7786

**Published:** 2015-10-01

**Authors:** Youssef Kort, Naziha Khammassi

**Affiliations:** 1 Faculté de médecine de Tunis, Service de médecine interne, Hôpital Razi, la Manouba 2010, Tunisie

**Keywords:** Syndrome de Gougerot Sjörgren, atteinte neurologique, atteinte du nerf hypoglosse, Gougerot Sjörgren syndrome, neurological involvement, hypoglossal nerve

## Image en medicine

L'atteinte neurologique périphérique étant plus fréquente que l'atteinte centrale au cours de Syndrome de Gougerot Sjogren (SGS) primitif. L'atteinte des paires crâniennes est rare dominée par l'atteinte de la cinquième, septième et huitième paire. Une atteinte isolée de la douzième paire est exceptionnelle et s'intègre généralement dans le cadre d'une atteinte multiple et récidivante des nerfs crâniens. Patiente de 62 ans sans antécédents pathologiques notables qui présentait depuis 6 ans un syndrome sec oculaire et buccal. L'examen notait une langue lisse, dépapillée et déviée vers la gauche à la protrusion (sans amyotrophie ni fasciculations) en rapport avec une atteinte de la douzième paire crânienne droite. Par ailleurs l'examen neurologique était sans anomalies notamment l'examen des autres paires crâniennes. L'examen ophtalmologique notait une kératite ponctuée superficielle bilatérale. Le bilan biologique était sans anomalies. La recherche d'anticorps antinucléaires était positive à 1/1280 de type moucheté de spécificité anti SSA et anti SSB. La recherche d'une cryoglobulinémie était négative. L'IRM cérébro-médullaire ainsi que l’électromyogramme étaient sans anomalies.. La biopsie des glandes salivaires accessoires était en faveur d'une sialadénite stade IV de Chisholm. Le diagnostic de SGS primitif a été retenu selon les critères européano américain. Aucun traitement spécifique n'a été préconisé pour l'atteinte neurologique étant donné son caractère asymptomatique. L'atteinte isolée du douze n'a à notre connaissance jamais été rapportée au cours du SGS. Les étiologies de l'atteinte du nerf hypoglosse sont essentiellement représentées par les tumeurs (malignes ou bénignes), les traumatismes et les malformations vasculaires du tronc cérébral.

**Figure 1:**
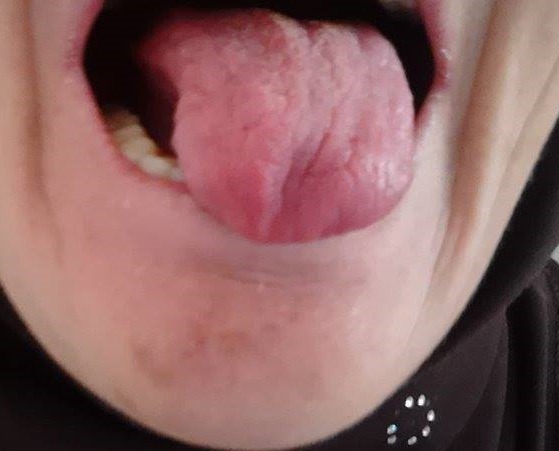
Déviation de la langue vers la gauche

